# Pharmacogenetics of the Primary and Metastatic Osteosarcoma: Gene Expression Profile Associated with Outcome

**DOI:** 10.3390/ijms24065607

**Published:** 2023-03-15

**Authors:** Alini Trujillo-Paolillo, Francine Tesser-Gamba, Maria Teresa Seixas Alves, Reynaldo Jesus Garcia Filho, Renato Oliveira, Antonio Sergio Petrilli, Silvia Regina Caminada Toledo

**Affiliations:** 1 Genetics Laboratory, Pediatric Oncology Institute (IOP/GRAACC), Federal University of Sao Paulo, Rua Botucatu, Vila Clementino, Sao Paulo 04023-062, SP, Brazil; 2 Department of Clinical and Experimental Oncology, Federal University of Sao Paulo, Rua Dr. Diogo de Faria, Vila Clementino, Sao Paulo 04037-003, SP, Brazil; 3 Department of Pathology, Federal University of Sao Paulo, Rua Botucatu, Vila Clementino, Sao Paulo 04023-062, SP, Brazil; 4 Department of Orthopedic Surgery and Traumatology, Federal University of Sao Paulo, Rua Borges Lagoa, Vila Clementino, Sao Paulo 04038-031, SP, Brazil; 5 Department of Thoracic Surgery, Federal University of Sao Paulo, Rua Napoleao de Barros, Vila Clementino 04024-002, SP, Brazil; 6 Pediatric Oncology Institute (IOP/GRAACC), Department of Pediatrics, Federal University of Sao Paulo, Rua Botucatu, Vila Clementino, Sao Paulo 04023-062, SP, Brazil

**Keywords:** osteosarcoma, metastatic osteosarcoma, pharmacogenetics, gene expression, prognostic markers, treatment response

## Abstract

Osteosarcoma (OS) is the most common malignant bone tumor in children and adolescents. In recent decades, OS treatment has reached a plateau and drug resistance is still a major challenge. Therefore, the present study aimed to analyze the expression of the genes related to pharmacogenetics in OS. The expression of 32 target genes in 80 paired specimens (pre-chemotherapeutic primary tumor, post-chemotherapeutic primary tumor and pulmonary metastasis) obtained from 33 patients diagnosed with OS were analyzed by the real-time PCR methodology. As the calibrators (control), five normal bone specimens were used. The present study identified associations between the OS outcome and the expression of the genes *TOP2A*, *DHFR*, *MTHFR*, *BCL2L1*, *CASP3*, *FASLG*, *GSTM3*, *SOD1*, ABCC1, *ABCC2*, *ABCC3*, *ABCC5*, *ABCC6*, *ABCC10*, *ABCC11*, *ABCG2*, *RALBP1*, *SLC19A1*, *SLC22A1*, *ERCC1* and *MSH2*. In addition, the expression of the *ABCC10*, *GGH*, *GSTM3* and *SLC22A1* genes were associated with the disease event, and the metastasis specimens showed a high expression profile of *ABCC1*, *ABCC3* and *ABCC4* genes and a low expression of *SLC22A1* and *ABCC10* genes, which is possibly an important factor for resistance in OS metastasis. Therefore, our findings may, in the future, contribute to clinical management as prognostic factors as well as possible therapeutic targets.

## 1. Introduction

Osteosarcoma (OS) is the most common primary malignant bone tumor in children and adolescents [1]. The overall survival probabilities have not improved during the last 30 years. Since then, the treatment has consisted of complete tumor resection after neoadjuvant chemotherapy, followed by adjuvant chemotherapy [2]. According to the EURAMOS-1 study results, the MAP regimen (Methotrexate, Adriamycin—doxorubicin, Platinol—cisplatin) must be considered the standard chemotherapy treatment for high-grade osteosarcoma [3].

Equivalent chemotherapeutic drug doses may lead to wide interpatient variability in treatment response, and it may be due to pharmacokinetic (Absorption, Distribution, Metabolism and Elimination—ADME) and pharmacodynamic (receptors and targets) differences in drugs [4]. The biological mechanisms involved in genetic variability are the differences in gene expression, epigenetics and genetic polymorphism [5]. Pharmacogenetics investigations have been explored in OS to understand the variability in treatment outcomes among patients [6,7]. Many pharmacogenomic studies have been conducted in OS and are beginning to yield insights into how to modify and improve chemotherapeutic approaches [8]. However, these studies have been focused merely on single nucleotide polymorphisms (SNPs) [9,10]. Moreover, a major priority in OS management is pulmonary metastasis, as this is the primary cause of death [11].

Therefore, the aim of the present study was to investigate the gene expression profile in a pharmacogenetic context, to the best of our knowledge, for the first time in paired OS specimens. The gene panel was designed based on MAP pharmacokinetic and pharmacodynamic modeling, as well as cell death and DNA damage repair processes that could be related to MAP response and OS tumorigenesis. The present study has investigated 32 genes involved in many processes, such as apoptosis—B-cell lymphoma 2 like 1 (*BCL2L1*), caspase 3 (*CASP3*) and Fas ligand (*FASLG*); cell cycle—cyclin-dependent kinase 1 (*CDK1*); damage recognition—high mobility group box 1 (*HMGB1*); DNA repair—excision repair cross-complementing 1 (*ERCC1*), excision repair cross-complementing 2 (*ERCC2*) and mutS homolog 2 (*MSH2*); detoxification—glutathione S-transferase (*GSTM1, GSTM3, GSTP1* and *GSTT1*) and superoxide dismutase 1 (*SOD1*); doxorubicin pathway—DNA topoisomerase II alpha (*TOP2A*); folate pathway—dihydrofolate reductase (*DHFR*) gamma-glutamyl hydrolase (*GGH*) and methylenetetrahydrofolate reductase (*MTHFR*); influx transport—solute carrier family (*SLC19A1*, *SLC22A1* and *SLC31A1*); and efflux transport—ATP binding cassette (*ABCB1*, *ABCC1*, *ABCC2*, *ABCC3*, *ABCC4*, *ABCC5*, *ABCC6*, *ABCC10*, *ABCC11* and *ABCG2*), ATPase copper transporting beta (*ATP7B*) and ralA binding protein 1 (*RALBP1*).

## 2. Results

### 2.1. Gene Expression Profile in Primary OS, Metastatic OS and Normal Bone

The expression of 32 target genes was investigated in OS and normal bone specimens. In some cases, this expression was different when comparing pre-chemotherapy with post-chemotherapy specimens, as well as in the primary and metastatic OS. The present investigation did not detect the expression of the *HMGB1* gene, neither in the OS nor in the normal bone specimens.

**Figure 1:**
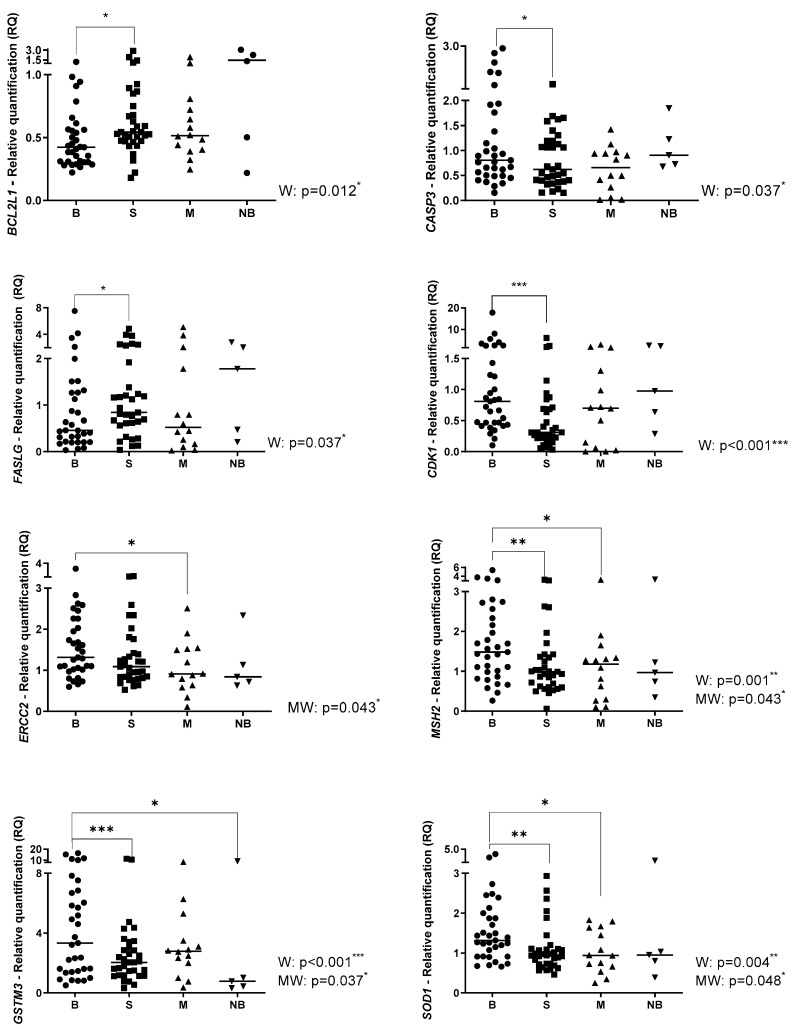

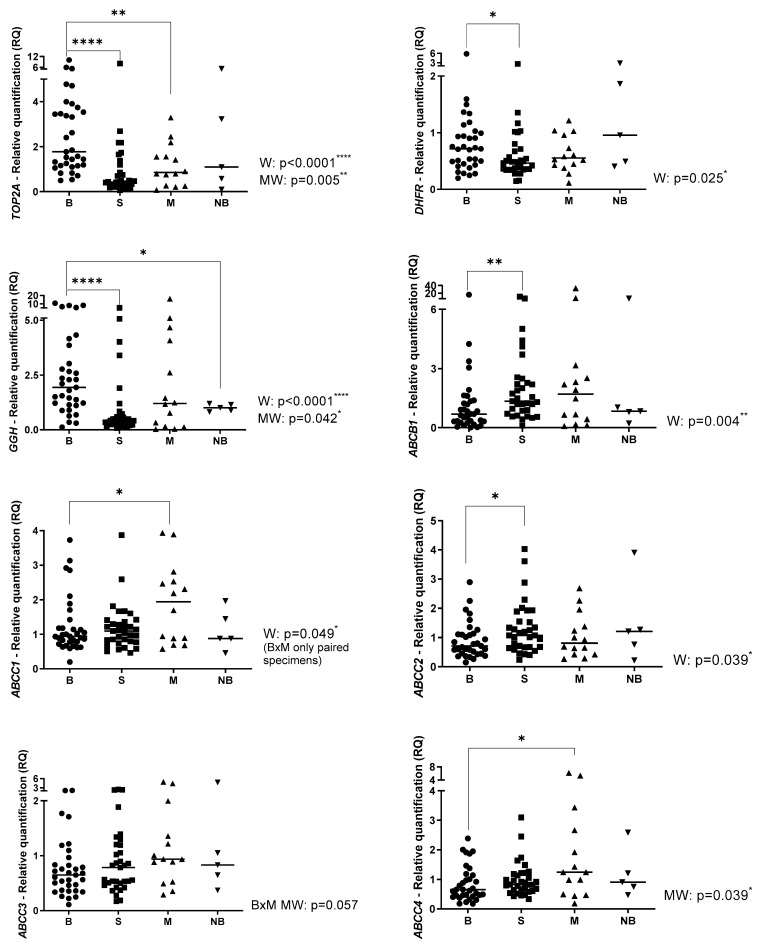

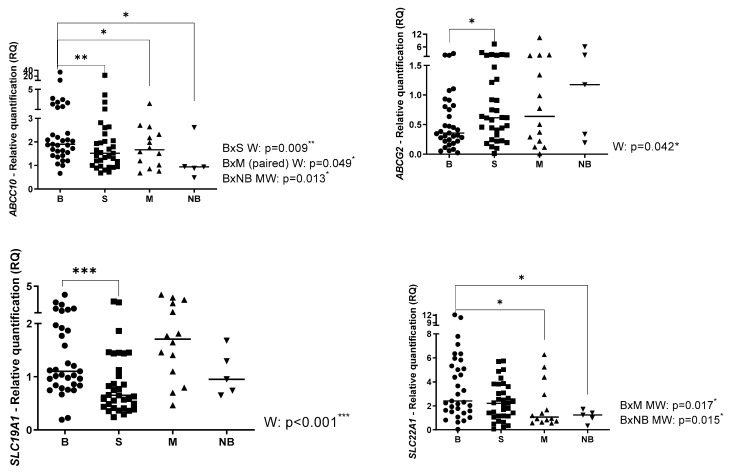
The relative quantification (RQ) in the pre-chemotherapy (B), post-chemotherapy (S), metastasis (M) and normal bone (NB) specimens. W: Wilcoxon Test; MW: Mann-Whitney Test. * *p* < 0.05; ** *p* < 0.01; *** *p* < 0.001; **** *p* < 0.0001.

The relative quantification (RQ) of the target genes with statistically significant results in all analyzed specimens: pre-chemotherapy (B), post-chemotherapy (S), metastasis (M) and normal bone (NB) are presented in Figure 1. The RQ of the target genes with non-statistically significant results is presented in the Supplementary Figure S1. Regarding the comparisons between primary OS and normal bone, evaluated by the Mann-Whitney test, higher expression of *GSTM3, GGH, ABCC10* and *SLC22A1* genes in OS was observed. (*p* = 0.037; *p* = 0.042; *p* = 0.013; and *p* = 0.015, respectively).

The comparisons between pre- and post-chemotherapy specimens were analyzed using the Wilcoxon test since all these samples were paired. The post-chemotherapy specimens presented higher expression of the *BCL2L1*, *FASLG*, *ABCB1*, *ABCC2* and *ABCG2* genes than the pre-chemotherapy specimens (*p* = 0.012; *p* = 0.037; *p* = 0.004; *p* = 0.039; and *p* = 0.042, respectively). The post-chemotherapy specimens presented lower expression of the *CASP3*, *CDK1*, *MSH2*, *GSTM3*, *SOD1*, *TOP2A*, *DHFR*, *GGH*, *ABCC10* and *SLC19A1* genes than the pre-chemotherapy specimens (*p* = 0.037; *p* < 0.001; *p* = 0.001; *p* < 0.001; *p* = 0.004; *p* < 0.0001; *p* = 0.025; *p* < 0.0001; *p* = 0.009; and *p* < 0.001, respectively).

The gene expression in metastasis specimens was compared with the pre-chemotherapy specimens using the Wilcoxon and Mann-Whitney test (paired and non-paired samples, respectively) because only 14 patients had developed metastasis. The metastasis specimens presented higher expression of *ABCC1*, *ABCC3* and *ABCC4* genes (*p* = 0.049; *p* = 0.057 (trend); and *p* = 0.039, respectively) and lower expression of *ERCC2, MSH2, SOD1, TOP2A, ABCC10* and *SLC22A1* genes (*p* = 0.043; *p* = 0.043; *p* = 0.048; *p* = 0.005; *p* = 0.049; and *p* = 0.017, respectively).

### 2.2. Gene Expression Profile Associated with Clinical Parameters

The clinical parameters were associated with gene expression, evaluated by the Mann-Whitney test (Table 1). Tumors from patients who were metastatic at diagnosis presented higher expression of *ABCB1*, *ABCC6*, *ABCC10*, *BCL2L1* and *SLC19A1* genes (*p* = 0.039; *p* = 0.048; *p* = 0.048; *p* = 0.026; and *p* = 0.010, respectively) than tumors from patients who were non-metastatic at diagnosis. Poor responders’ tumors presented higher expression of *ERCC1* and *TOP2A* genes (*p* = 0.021 and *p* = 0.036, respectively) and lower expression of *ABCC3*, *FASLG* and *SLC22A1* genes (*p* = 0.031; *p* = 0.017; and *p* = 0.014, respectively) than good responders’ tumors. The sizes of tumors resected in surgery were also associated with the expression of the investigated genes. Large tumors of 12 cm or more presented lower expression of *ABCG2*, *CASP3* and *MSH2* genes (*p* = 0.027; *p* = 0.033; and *p* = 0.045, respectively) than small tumors. The local control was conducted with surgery that could be either conservative or an amputation. Tumors from patients who underwent amputation presented higher expression of *ABCC11*, *DHFR*, *ERCC1*, *GSTM3*, *SLC19A1* and *TOP2A* genes (*p* = 0.002; *p* = 0.007; *p* = 0.0042; *p* = 0.022; *p* = 0.002. and *p* = 0.010, respectively) and lower expression of *FASLG*, *MTHFR* and *SLC22A1* genes (*p* < 0.0001; *p* = 0.003; and *p* = 0.024, respectively) than tumors from patients who underwent conservative surgery. In the relapse analyses, one patient was excluded because he had disease progression during the first treatment and died before he reached remission. Tumors from patients who relapsed presented higher expression of *TOP2A* (*p* = 0.038) and lower expression of *ABCC3*, *ABCC5* and *FASLG* genes (*p* = 0.026; e *p* = 0.051; *p* = 0.050, respectively) than patients who not relapsed. Regarding the *ABCC5* result, this was only a trend of statistical significance.

### 2.3. Gene Expression Profile Associated with OAS and EFS

**Figure 2:**
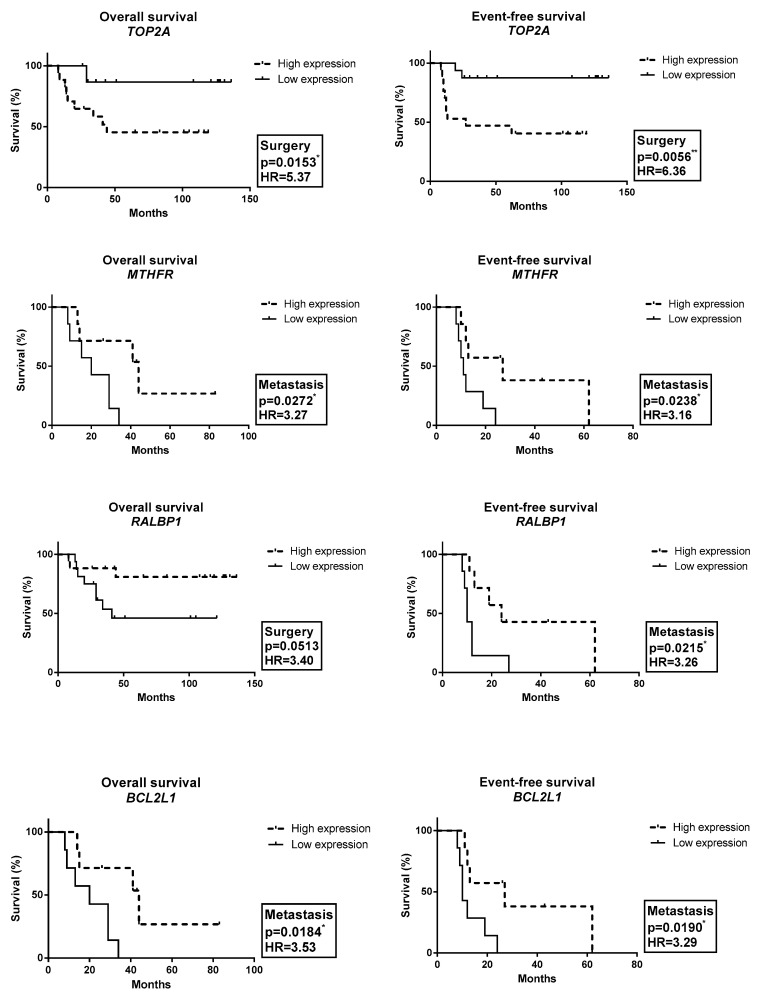

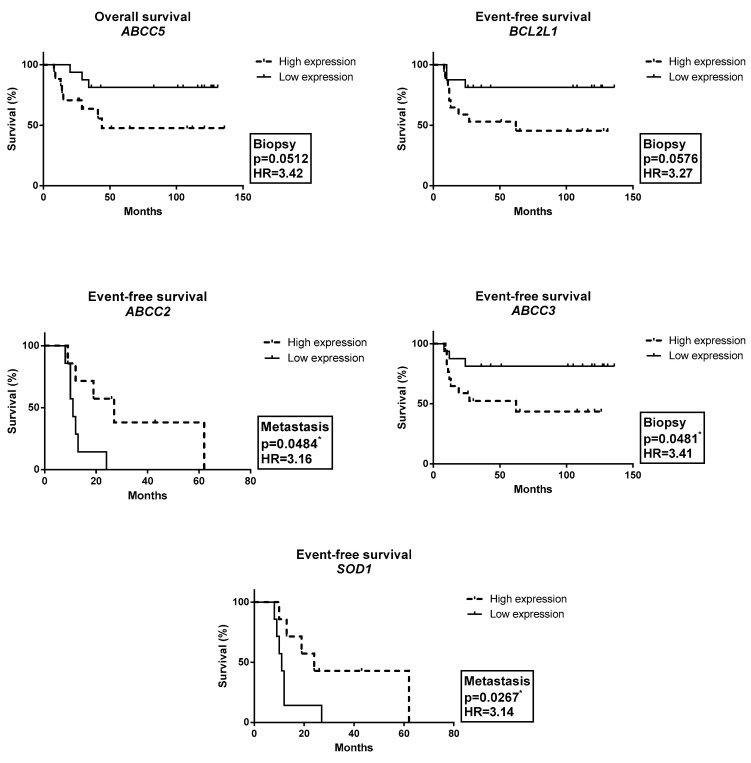
Association between gene expression and survival in OS. Pre-chemotherapy (Biopsy); Post-chemotherapy (Surgery); and Metastasis (M). * *p* < 0.05; ** *p* < 0.01.

As shown in Figure 2, patients with high expression of the *ABCC5* and *BCL2L1* genes in the pre-chemotherapy biopsy had a trend towards worse OAS (*p* = 0.051; HR = 3.42) and EFS (*p* = 0.058; HR = 3.27), respectively, compared with patients with low expression of the *ABCC5* and *BCL2L1* genes. Moreover, patients with high expression of the *ABCC3* gene in the pre-chemotherapy biopsy had worse EFS compared with patients with low expression of the *ABCC3* gene (*p* = 0.048; HR = 3.41). Patients with high expression of the *TOP2A* gene in the post-chemotherapy specimens had worse OAS (*p* = 0.015; HR = 5.37) and EFS (*p* = 0.005; HR = 6.36) compared with patients with low expression of the *TOP2A* gene. Furthermore, patients with low expression of the *RALBP1A* gene in the post-chemotherapy specimens had a trend towards worse OAS compared with patients with high expression of the *RALBP1* gene (*p* = 0.051; HR = 3.40). Patients with low expression of the *BCL2L1* and *MTHFR* genes in the metastasis had worse OAS (*p* = 0.018, HR = 3.53; and *p* = 0.027, HR = 3.27, respectively) and worse EFS (*p* = 0.019, HR = 3.29; and *p* = 0.024, HR = 3.16, respectively) compared with patients with high expression of the *BCL2L1* and *MTHFR* genes. Moreover, patients with low expression of the *ABCC2, RALBP1* and *SOD1* genes in the metastasis specimens had worse EFS (*p* = 0.048, HR = 3.16; *p* = 0.022, HR = 3.26; and *p* = 0.027, HR = 3.14, respectively) compared with patients with high expression of the *ABCC2, RALBP1* and *SOD1* genes.

## 3. Discussion

The expression of genes analyzed in the present study was investigated for the first time in paired OS specimens. When comparing OS samples obtained pre-and post-treatment, as well as from metastases, we detected different levels of expression of the selected genes. Moreover, this study showed that the genes related to a treatment response could be associated with OS tumorigenesis.

TOP2A is a target for several anticancer agents, such as doxorubicin, and a variety of mutations in this gene have been associated with the development of drug resistance. This nuclear enzyme is involved in processes such as chromosome condensation, chromatid separation and the relief of torsional stress that occurs during DNA transcription and replication [12]. A meta-analysis showed that high *TOP2A* expression is associated with a worse prognosis in many types of cancer [13]. In OS, the presence of *TOP2A* amplification tends to relate to a worse overall survival rate [14]. The present study showed an association between high expression of *TOP2A* and poor response, amputation and relapse. Moreover, high expression was also associated with worse OAS and EFS.

*DHFR*, *GGH* and *MTHFR* are genes involved in the methotrexate pathway and response [15]. Methotrexate resistance in human OS cells is associated with an amplification and/or overexpression of its target, the *DHFR* [6]. We observed that patients who underwent amputation presented metastasis with higher *DHFR* expression than patients who underwent conservative surgery. Increased levels of *GGH* led to a decreased accumulation of polyglutamated MTX and MTX resistance [16]. In OS, the ratio between the patients and the controls for the polymorphisms GGH_452T/C, GGH_401T/C and GGH_16T/C was greater than 1.5. The GGH_401C/T variant enhanced promoter activity, increasing protein expression [9]. In the present study, it was observed that OS presented higher *GGH* expression than in normal bone. The rs1801133 polymorphism of the *MTHFR* has been the most frequently studied in OS and leads to a C to T substitution, resulting in decreased enzymatic activity. In OS, the TT genotype was significantly associated with toxicity [15]. However, we observed that low expression in OS metastasis was associated with worse OAS and EFS. Moreover, the patients who underwent amputation had lower *MTHFR* expression in the primary tumor compared with the patients who underwent conservative surgery.

Regarding apoptosis, *BCL2L1*, *CASP3* and *FASLG* were investigated in the present study. The longer isoform of *BCL2L1* acts as an apoptotic inhibitor and the shorter isoform acts as an apoptotic activator [17]. We found that high *BCL2L1* expression in the primary tumor was associated with metastasis at diagnosis and a worse EFS. Nevertheless, in the metastatic tumor, low expression was associated with worse OAS and EFS. This discrepancy in our results could be explained by the theory that the primary tumor expresses the longer isoform (anti-apoptosis) and a metastatic tumor expresses the shorter isoform (pro-apoptosis), since chemotherapy drugs stimulate the production of the shorter isoform [18]. The G allele of the variant rs2720376, linked with lower *CASP3* expression, was associated with a lower EFS in OS [10]. In this study, low *CASP3* expression was associated with large tumors. The variant rs763110, linked to a lower FasL expression, was associated with a lower EFS in OS [10]. We found that low *FASLG* expression was associated with a poor response, amputation and relapse.

Regarding genes related to detoxification of the chemotherapeutic drugs, *GSTM1* and *GSTP1* presented no association with the outcome in OS. The polymorphism in *GSTM3* (AA versus BB) has been associated with OS risk [19]. The present study observed an association between high *GSTM3* expression and amputation. Moreover, OS presented higher *GSTM3* expression than normal bone. The *GSTM3* polymorphism could confer different efficiencies in the metabolism of carcinogens and has been shown to modulate various cancers’ risk [20]. The null *GSTT1* genotype was associated with OS risk [19]. Resistant cell lines of OS showed lower *SOD1* expression than their parental cells [21]. We found that metastasis presented lower *SOD1* expression than the primary tumor, and patients with low expression of the *SOD1* gene in the metastasis had worse EFS.

Genes involved in the repair of DNA adducts induced by cisplatin, which thereby influence cisplatin efficacy, have been investigated by the largest number of studies on OS [7]. ERCC1 positivity has presented an association with poor EFS and OAS in OS [22]. The present study showed an association between high expression and poor response and amputation. The *ERCC2* rs1799793 polymorphism was related to the high risk of OS development [23]. This study showed that metastasis presented lower *ERCC2* expression than primary tumors. We found that low *MSH2* expression was associated with large primary tumors. Metastasis specimens presented lower expression than primary tumors. In OS, the variant rs4638843 in *MSH2* was associated with a worse EFS [10]. Moreover, a wide investigation of childhood cancers found germline mutations of *MSH2* in OS [24]. Taken together, our results showed that metastasis in OS presents low expression of the *ERCC2* and *MSH2* genes compared with pre-chemotherapy biopsy, which could be related to decreased ability to repair DNA damage in metastasis, possibly resulting in genetic alterations accumulation and more aggressive cancer [25].

High efflux transporter gene expression and low influx transporter gene expression are the main resistance mechanisms related to cisplatin, doxorubicin and methotrexate, in vitro. Moreover, many polymorphisms in these genes have been related to treatment response in OS [6,7,26,27,28]. The present study, for the first time, investigated the expression of transporter genes in paired specimens. The results showed that the tumor biopsy presented high *ABCC6* and *ABCC10* expression, and metastasis presented high *ABCB1* expression when metastasis was present at diagnosis. The patients with high *ABCC3* and *ABCC5* expression in biopsy presented worse EFS and OAS, respectively, and patients with low *ABCC2* expression in metastasis presented worse EFS.The patients with low *RALBP1* expression in surgery and metastasis presented worse OAS and EFS, respectively.

OS presented higher *ABCC10* and *SLC22A1* expression than normal bone. However, low *SLC22A1* expression was associated with a poor response and amputation, probably due to its influx function. This is the first investigation regarding *SLC22A1* and OS. *SLC22A1* could be activated by miR-21, which is overexpressed in OS and was associated with tumorigenesis [29]. Moreover, metastasis presented higher *ABCC1*, *ABCC3* and *ABCC4* expression and lower *SLC22A1* and *ABCC10* expression than the primary tumor. This pattern could contribute to the lower intracellular concentration of the chemotherapeutic drugs. Consequently, it could contribute to the mechanism of resistance in metastasis, which is the main cause of death in OS patients. Therefore, with the knowledge of the metastasis profile, it is possible to develop new strategies for these patients. CBT-1^®^ is an adjunct to chemotherapy in all cancer types with multi-drug resistance. Eight clinical trials are evaluating CBT-1^®^ in patients with many cancer types, such as acute myelogenous leukemia, breast, non-Hodgkin’s lymphoma, Hodgkin’s disease, lung, and sarcoma [30,31]. Moreover, CBT-1® was able to revert the ABCB1/ABCC1-mediated resistance against doxorubicin in OS cell lines [32]. In the future, it could be interesting to evaluate CBT-1^®^ in metastatic OS patients 

In conclusion, the present study identified associations between OS outcome and expression of the genes *TOP2A*, *DHFR*, *MTHFR*, *BCL2L1*, *CASP3*, *FASLG*, *GSTM3*, *SOD1*, *ABCB1*, *ABCC2*, *ABCC3*, *ABCC5*, *ABCC6*, *ABCC10*, *ABCC11*, *ABCG2*, *RALBP1*, *SLC19A1*, *SLC22A1*, *ERCC1* and *MSH2*. In addition, the pre-chemotherapy biopsy from OS patients had higher gene expression of *ABCC10*, *GGH*, *GSTM3* and *SLC22A1* compared with bone specimens obtained from healthy subjects, and the metastasis specimens showed a high expression profile of *ABCC1*, *ABCC3* and *ABCC4* and low expression of *SLC22A1* and *ABCC10*, which is possibly an important factor for resistance in OS metastasis. In summary, we found that the expression of genes related MAP pharmacokinetic and pharmacodynamic modeling, as well as cell death and DNA damage repair processes are associated with OS tumorigenesis and MAP response in OS patients. Therefore, in the future, our findings may contribute to clinical management as prognostic markers and also as possible therapeutic targets.

## 4. Materials and Methods

### 4.1. Patients and Specimens

We investigated 80 paired specimens obtained from 33 patients with diagnoses of OS. These patients were admitted to the Pediatric Oncology Institute (IOP/GRAACC/UNIFESP) between 2006 and 2016. The average age at diagnosis was 13 years old. Of 33 OS patients, 14 patients presented pulmonary metastasis. Thus, we investigated 33 biopsy specimens (pre-chemotherapy), 33 surgery specimens (post-chemotherapy) and 14 pulmonary metastasis specimens. Five normal bone tissues were used as a control; they were obtained from orthopedic surgeries of five healthy individuals that underwent trauma and did not present either genetic disorders or bone diseases. This study had the Research Ethics Committee approval from the Federal University of Sao Paulo (N° 0189/2016), and all patients agreed to participate by informed consent. All patients were treated following the GLATO (Grupo Latino Americano de Tratamento de Osteossarcoma—Latin American Group of Osteosarcoma Treatment) protocol of 2006, which is based on high doses of cisplatin, doxorubicin and methotrexate. All clinical data are summarized in Table 2.

### 4.2. Gene Expression (qRT-PCR)

The expression of 32 genes involved with pharmacogenetics was measured by a quantitative reverse transcription PCR (qRT-PCR). All frozen tissues were submitted to an RNA extraction using TRIzol^®^ Reagent (Thermo Fisher Scientific, Waltham, MA, USA). The cDNA was synthesized using SuperScript^®^ Vilo™ Master Mix (Invitrogen, Waltham, MA, USA). The qRT-PCR was performed in triplicate using TaqMan^®^ Gene Expression Assays (Thermo Fisher Scientific, Waltham, MA, USA) (Table 3). The *ACTB* and *GAPDH* genes were used as endogenous controls. Normal bone was used as a calibrator.

### 4.3. Statistical Analyses

Data analyses were performed using GraphPad Prism version 6.0 for Windows (GraphPad Software, San Diego, CA, USA). The gene expression measured by relative quantification was compared using nonparametric tests: the Wilcoxon and Mann-Whitney tests. The overall survival (OAS) and event-free survival (EFS) were calculated using the Kaplan-Meier method and the survival curves were compared using the log-rank test. The time of relapse was considered the time from the OS diagnosis until the relapse event. For OAS and EFS analyses, the median value of each gene and specimen type (biopsy, surgery or metastasis) was the cut-off that defined high or low expression. Statistical significance was considered when *p* < 0.05.

## Figures and Tables

**Table 1:** Association between gene expression and clinical parameters.

Clinical Parameter	Gene	Expression	Specimen	*p*
Metastasis at diagnosis * vs. Non-metastasis at diagnosis	*ABCB1*	↑	M	0.039
	*ABCC6*	↑	B	0.048
	*ABCC10*	↑	B	0.048
	*BCL2L1*	↑	S	0.026
	*SLC19A1*	↑	S	0.010
*** Poor responder * vs. Good responder	*ABCC3*	↓	S	0.031
	*ERCC1*	↑	B	0.021
	*FASLG*	↓	S	0.017
	*SLC22A1*	↓	B	0.014
	*TOP2A*	↑	S	0.036
Large tumor (>12 cm) * vs. Small tumor	*ABCG2*	↓	S	0.027
	*CASP3*	↓	B	0.033
	*MSH2*	↓	B	0.045
Amputation * vs. Conservative surgery	*ABCC11*	↑	M	0.042
	*DHFR*	↑	M	0.007
	*ERCC1*	↑	M	0.042
	*FASLG*	↓	S	<0.0001
		↓	M	0.042
	*GSTM3*	↑	S	0.022
	*MTHFR*	↓	S	0.003
	*SLC19A1*	↑	B	0.002
		↑	S	0.016
	*SLC22A1*	↓	B	0.024
	*TOP2A*	↑	S	0.010
Relapse * vs. Non-relapse	*ABCC3*	↓	M	0.026
	*ABCC5*	↓	M	0.051 **
	*FASLG*	↓	S	0.041
	*TOP2A*	↑	S	0.038

Higher gene expression (↑); Lower gene expression (↓); Pre-chemotherapy—Biopsy (B), Post-chemotherapy—Surgery (S); Metastasis (M). * Clinical parameter associated with higher or lower gene expression. Per example, patients metastatic at diagnosis had higher gene expression of *ABCB1* gene in the metastasis specimens compared with the non-metastatic at diagnosis patients. ** Trend of statistical significance. *** Poor responder: <90% of necrosis post-neoadjuvant therapy.

**Table 2:** Clinical features of OS patients.

Clinical Features	N (%)
**Gender**	
Male	64%
Female	36%
**Age**	
≤13	42%
>13	58%
**Metastasis at diagnosis**	
Yes	33
No	67
**Primary site**	
Femur	67
Tibia	24
Humerus	9
**Surgery**	
Conservative	85
Amputation	15
**Histology**	
Mixed	24
Osteoblastic	55
Condroblastic	9
Fibroblastic	3
Telangiectatic	3
Not identified	6
**Tumor size**	
<12 cm	55
≥12 cm	42
Not identified	3
**Grade of tumor necrosis**	
≤90%	49
>90%	45
Not identified	6
**Pulmonary metastasis**	
Yes	42
No	57
**Relapse**	
Yes	33
No	67
**Death**	
Yes	33
No	67

N: Number of patients.

**Table 3:** TaqMan^®^ Gene Expression Assays.

Function	Gene	Assay
Apoptosis	*BCL2L1*	Hs00236329_m1
	*CASP3*	Hs00234387_m1
	*FASL*	Hs00181226_g1
Cell cycle	*CDK1*	Hs00938777_m1
Damage recognition	*HMGB1*	Hs01923466_g1
DNA repair	*ERCC1*	Hs01012158_m1
	*ERCC2*	Hs00361161_m1
	*MSH2*	Hs00953527_m1
Detoxification	*GSTM1*	Hs01683722_gH
	*GSTM3*	Hs00356079_m1
	*GSTP1*	Hs00943350_g1
	*GSTT1*	Hs02512069_s1
	*SOD1*	Hs00533490_m1
Doxorubicin pathway	*TOP2A*	Hs01032137_m1
Endogenous	*ACTB*	Hs01060665_g1
	*GAPDH*	Hs02758991_g1
Folate pathway	*DHFR*	Hs00758822_s1
	*GGH*	Hs00914163_m1
	*MTHFR*	Hs01114487_m1
Transport	*ABCB1*	Hs00184500_m1
	*ABCC1*	Hs01561502_m1
	*ABCC2*	Hs00166123_m1
	*ABCC3*	Hs00978452_m1
	*ABCC4*	Hs00988717_m1
	*ABCC5*	Hs00981089_m1
	*ABCC6*	Hs00184566_m1
	*ABCC10*	Hs01056200_m1
	*ABCC11*	Hs01090758_m1
	*ABCG2*	Hs01053790_m1
	*ATP7B*	Hs00163739_m1
	*RALBP1*	Hs01034984_g1
	*SLC19A1*	Hs00953344_m1
	*SLC22A1*	Hs00427552_m1
	*SLC31A1*	Hs00977266_g1

## Data Availability

The data presented in this study are available on request from the corresponding author.

## Supplementary Materials

The following supporting information can be downloaded at: https://www.mdpi.com/article/10.3390/ijms24065607/s1.
