# Supplementary material for: Pharmacogenetics of the Primary and Metastatic Osteosarcoma: Gene Expression Profile Associated with Outcome
Source: Int J Mol Sci. 2023 Mar 15;24(6):5607. doi: 10.3390/ijms24065607 (PMC10059037; doi:10.3390/ijms24065607)
Supplement: Supplementary file 1 [file ijms-24-05607-s001.zip › ijms-2154318-supplementary.pdf]

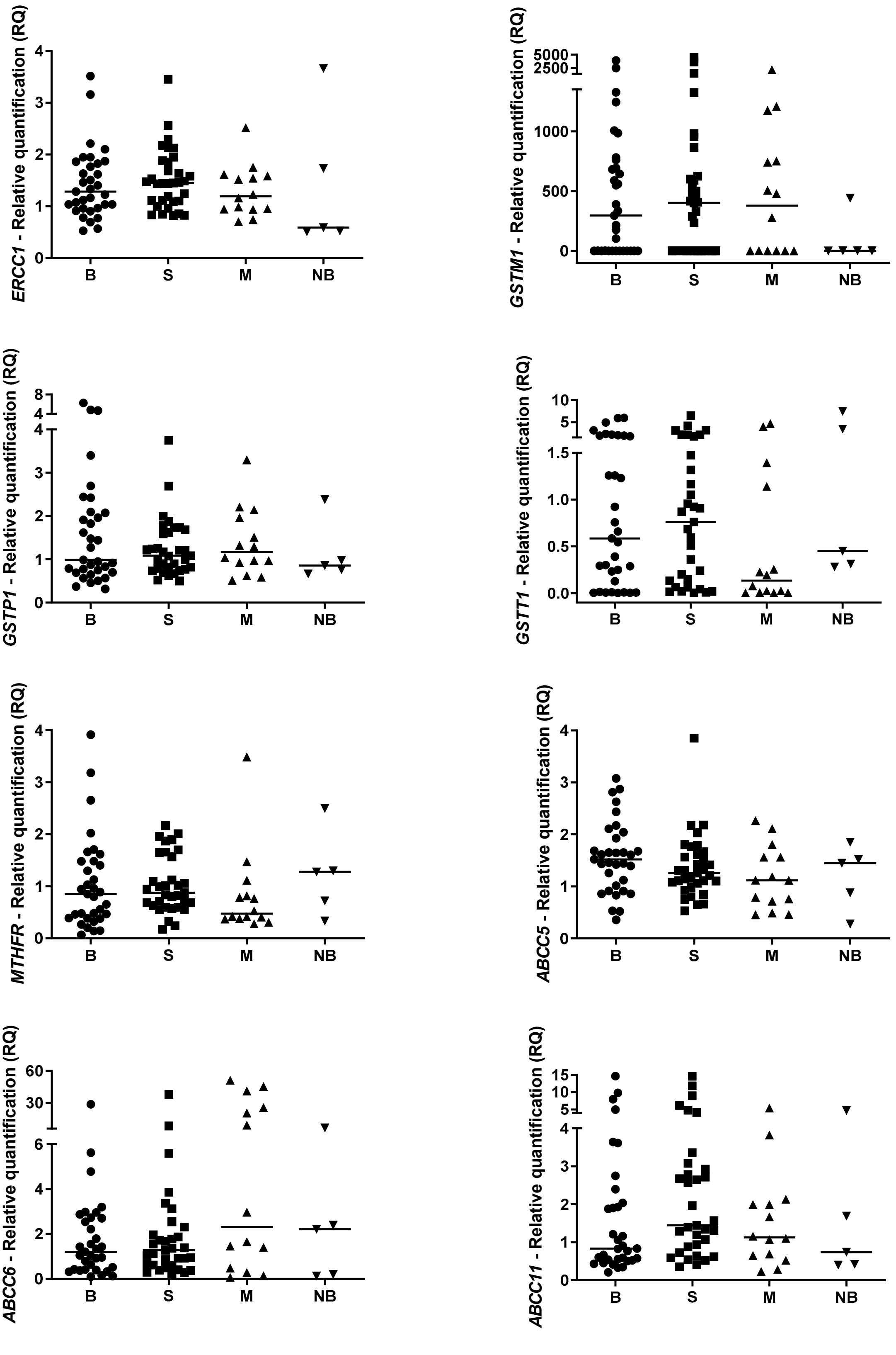
ERCC1 - Relative quantification (RQ)
GSTM1 - Relative quantification (RQ)
GSTP1 - Relative quantification (RQ)
GSTT1 - Relative quantification (RQ)
MTHFR - Relative quantification (RQ)
ABCC5 - Relative quantification (RQ)
ABCC6 - Relative quantification (RQ)
ABCC11 - Relative quantification (RQ)
B
S
M
NB

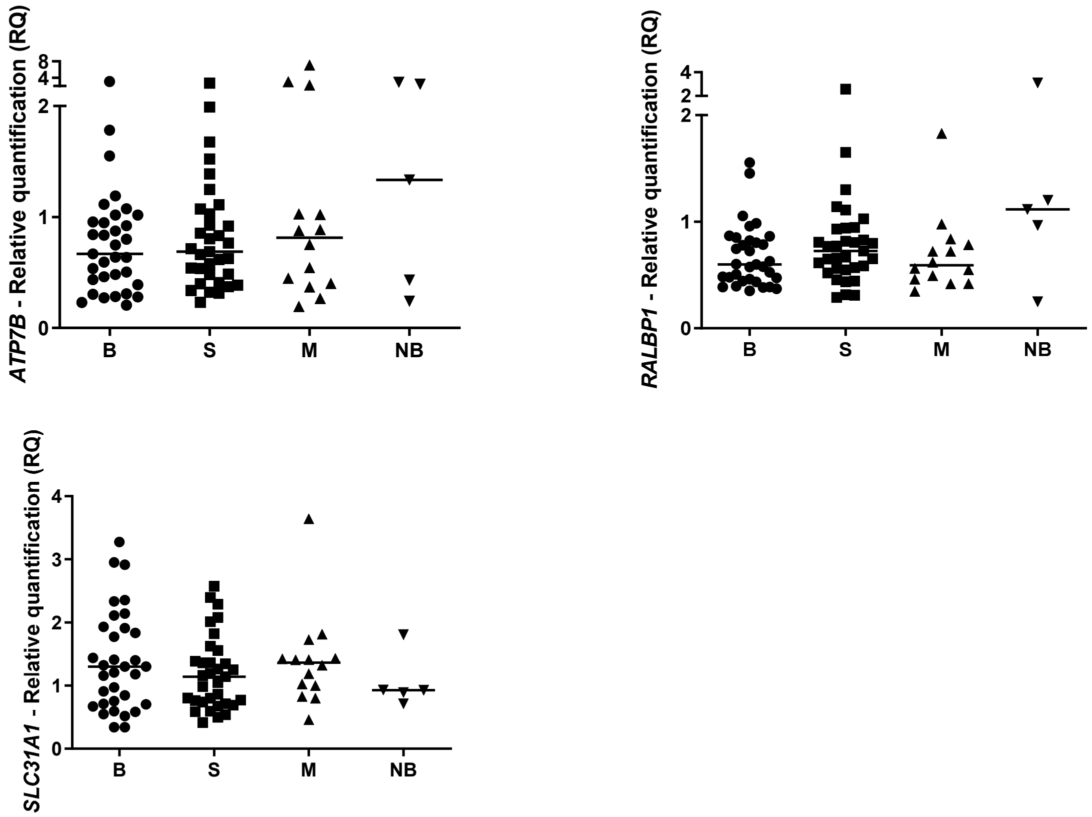

Supplementary Figure S1. The relative quantification (RQ) of the target genes with not statistically significant results in all analyzed specimens, pre-chemotherapy (B), post-chemotherapy (S), metastasis (M) and normal bone (NB).
